# The Association between Serum Bilirubin Level and Electrochemical Skin Conductance in Chinese Patients with Type 2 Diabetes

**DOI:** 10.1155/2018/6253170

**Published:** 2018-03-07

**Authors:** Fei Mao, Xiaoming Zhu, Bin Lu, Yiming Li

**Affiliations:** ^1^Department of Endocrinology and Metabolism, Huashan Hospital, Fudan University, Shanghai, China; ^2^Department of Endocrinology and Metabolism, Jing'an District Center Hospital of Shanghai, Shanghai, China

## Abstract

Bilirubin is an antioxidant and plays a protective role against cardiovascular and microvascular disease. The aim of this study is to explore the possible protective effect of bilirubin on small nerve function. A total of 265 Chinese patients with type 2 diabetes mellitus (T2DM) were enrolled in the study. Both SUDOSCAN and other traditional diabetic neuropathy examinations including neuropathy symptom score (NSS), the neuropathy disability score (NDS) and Michigan Neuropathy Screening Instrument (MNSI) scores were performed in all patients with T2DM. Blood bilirubin levels were tested in the study. Spearman correlation analysis and multivariate regression analysis were performed to determine the relation between bilirubin level and hands and feet ESC values. Spearman correlation analysis demonstrated a correlation between total bilirubin and ESC levels including hands (*r* = 0.165, *P* < 0.05) and feet (*r* = 0.122, *P* < 0.05) as well as between UCBil and ESC levels including both hands (*r* = 0.172, *P* < 0.05) and feet (*r* = 0.175, *P* < 0.05). Multivariate regression linear analyses showed both total bilirubin and UCBil level were independently associated with hands and feet ESC levels. All these results suggested a positive association between bilirubin level and ESC level, indicating a possible protective role of bilirubin in peripheral small nerve dysfunction of type 2 diabetes mellitus.

## 1. Introduction

Bilirubin, as the end product of heme catabolism, has been regarded merely as a waste with potential toxicity for a long time until year 1987 when it was identified as an antioxidant of possible physiological importance [1]. Both autonomic and peripheral nerve functions were found to be correlated with serum bilirubin after autonomic and peripheral nerve functions were examined in a group of patients with primary biliary cirrhosis by using standard cardiovascular reflex tests and peripheral nerve conduction studies [2].

In the year 1996, low level of serum bilirubin concentration was first reported to be closely related to increased chronic vascular disease (CVD) risk factors in a large Chinese population [3]. Later in other studies, bilirubin, recognized as an antioxidant and anti-inflammatory metabolic molecule, has been further proved to be significantly inversely correlated with the incidence of coronary artery disease [1, 4, 5], stroke [6], and peripheral artery disease [6, 7] independent of conventional risk factors in general population and higher levels of bilirubin would reduce susceptibility to vascular diseases. A higher level of serum total bilirubin (TBil) level was found to be associated with a lower incidence of T2DM [8], and also low TBil level was associated with diabetic microvascular complications including diabetic retinopathy [9–13] and microalbuminuria [9, 14] as well as subclinical atherosclerosis such as an increased risk of amputation [10].

However, few studies have focused on the relationship between TBil levels and diabetic peripheral neuropathy (DPN) in T2DM patients. DPN is one of the common chronic complications of type 2 diabetes, with an estimated prevalence of 50%–70% depending on different diagnostic methods [15]. Despite many different factors including oxidative stress and inflammation caused by long-term exposure to hyperglycemia that have already been implicated in its pathogenesis, the underlying mechanism of DPN is still not fully understood. Evaluation of sweating has the appeal of quantifiable noninvasive determination of the integrity of the peripheral autonomic nervous system and can now be accomplished rapidly at point of care clinics with the determination of ESC level [16]. The SUDOSCAN, a new screening device for early detection of DPN, has been developed based on different electrochemical principles (reverse iontophoresis and chronoamperometry) to measure sudomotor function through ESC level of hands and feet [17–19]. It provides a more practical and precise performance profile as a tool for the early detection of peripheral small nerve dysfunction. Low ESC level produced by the SUDOSCAN indicated an impairment of small nerve in the early stage of T2DM.

In this study, we aim to evaluate the association between serum bilirubin concentration and the extent of peripheral small nerve dysfunction by using ESC level detected by the SUDOSCAN in Chinese patients with type 2 diabetes.

## 2. Materials and Methods

### 2.1. Study Population

The study was conducted in Huashan Hospital, Shanghai, from September 2014 to September 2015. The ethics committee of Huashan Hospital approved the study. Voluntary outpatients diagnosed with type 2 diabetes between 18 and 80 years of age, with or without symptoms of neuropathy, were continually enrolled in the study. The aims of the study were explained to the participants, and the fact that they could withdraw from the study at any time during the study was informed to them. Exclusion criteria included undiagnosed hyperglycemia, type l diabetes mellitus (T1DM), treatment with drugs that could have an effect on the sympathetic system such as beta blockers and antineoplastic drugs, implantation of electrical implantable devices, history of seizures or epilepsy, lumbar sciatic nerve lesion, severe varices of the lower limbs, other metabolic diseases including thyroid disease or vitamin B12 deficiency, and any other advanced systemic condition including severe hepatic and renal dysfunction as reported by our groups before [20].

### 2.2. Physical Examination

One trained nurse examined all patients and recorded the results. Basic physical characteristics (height, weight, and waist and hip circumference) were measured using standard methods, and body mass index (BMI) and waist-hip ratio (WHR) were calculated. Blood pressure was recorded in the supine position following 5 min of rest. Medical history of diabetes was recorded completely for each patient.

### 2.3. Laboratory Examination

Blood samples were collected for plasma HbA1c level determined by high-pressure liquid chromatography and liquid enzymatic assay. Blood samples were also collected for serum aspartate aminotransferase (AST), alanine aminotransferase (ALT), total cholesterol (TC), high-density lipoprotein cholesterol (HDL-C), triglycerides (TG), low-density lipoprotein cholesterol (LDL-C), serum total bilirubin (TBiL) level, conjugated bilirubin level (CBil), and unconjugated bilirubin (UCBil) level which were measured by using an automatic analyzer (AU640; Olympus Corporation, Tokyo, Japan) after at least 8 hours of fasting.

### 2.4. SUDOSCAN Test Procedure

The SUDOSCAN device is composed of two sets of electrodes for the feet and hands, both of which are connected to a computer for recording and data analysis. The procedure of measurement was described in previously published studies in detail. The device can measure electrochemical skin conductance (ESC) values expressed in micro-Siemens (*μ*S) for the hands and the feet (both right and left sides). Higher ESC level stands up for less possibility of neuropathy.

The SUDOSCAN detection was accepted by all the subjects without any complaint of discomfort, and no safety events were ever reported as described in early studies [20].

As for the diagnostic criteria of the SUDOSCAN test, we used 60 *μ*S of mean foot ESC as the cut-off for the diagnosis of DPN according to previous studies [19, 21].

### 2.5. Peripheral Neuropathy Examination

Symptoms and signs of lower limbs were recorded, respectively. The assessments of the DPN were performed by one expert nurse using three different questionnaires including the neuropathy symptom score (NSS) [22], the neuropathy disability score (NDS) [22], and Michigan Neuropathy Screening Instrument (MNSI score) [23, 24] as have been used before in our group. A composite score was calculated separately for neuropathic symptoms using the NSS score questionnaire and for clinical examination using the NDS score. Neurological symptoms and signs based on the neuropathy symptom score (NSS) and the neuropathy disability scores (NDS) were evaluated. Neurological symptoms included burning, numbness, tingling, fatigue, cramping, or aching, and neurological signs included vibration sense (128 Hz tuning fork at the great toe), pain (pinprick sensation at the great toe), temperature sensation (cold tuning fork at the great toe), and ankle reflex.

According to previous standards, an NSS score of 3-4 points was considered a mild neuropathy symptom, 5-6 points as medium neuropathy symptom, and 7–9 points as severe neuropathy symptom. The results of the NDS score were divided into three classes as well. A patient with an NDS of 3–5 points was considered with mild neuropathy signs, 6–8 points as medium neuropathy signs, and 9-10 points as severe neuropathy signs. The golden diagnosis standard of DPN is dependent on both the NSS and the NDS scores (with an NDS score of ≥6 or an NDS score of 3–5 associated with an NSS score of ≥5) as has been used before in our previous study.

The MNSI score consists of two parts: the appearance of the feet (deformity, dry skin, callus, infection, or fissures) and examination of foot ulceration, ankle reflex, and vibration perception with a 128 Hz tuning fork. The evaluation of each parameter was made at both sides with a maximum score of 8 points.

### 2.6. Statistical Analysis

Results of continuously measured characteristics were expressed as mean ± standard deviation (SD), and distributions of categorical variables are expressed as percentages and absolute numbers. Analysis of variance was used to compare mean differences of clinical factors between the DPN group and the non-DPN group, and *χ*^2^ analyses were used to assess differences between categorical variables.

Spearman correlation analysis was performed on both hand and foot ESC values and bilirubin level including total bilirubin, conjugated and unconjugated bilirubin levels. The association between hand ESC values as well as foot ESC values with bilirubin levels was analyzed by multivariable linear regression models adjusted for age, BMI, and SBP. For all multivariable analyses, a *P* value of <0.05 was considered statistically significant. All analyses were performed using Stata v11.1 (Stata Corp., College Station, TX).

## 3. Results

We studied a total of 265 patients with type 2 diabetes mellitus (163 males and 102 females) in the study. Among those 265 patients with type 2 diabetes, mean age is 59.64 ± 12.83 years, mean duration of type 2 diabetes is 9.20 ± 7.84 years, and mean HbA1C% level is 8.67 ± 2.18%. Clinical and biochemical characteristics of the 265 patients are described in Table 1. We divided the patients into two different groups by DPN using SUDOSCAN results as diagnostic criteria. As indicated in Table 2, both hand and foot ESC levels are much lower in both DPN diagnosed by the MNSI and NSS/NDS scores (*P* < 0.01). T2DM patients in the study with DPN diagnosed by the SUDOSCAN had a longer duration of T2DM (*P* < 0.05), higher WHR level (*P* < 0.05), higher NSS score (*P* < 0.001), higher NDS score (*P* < 0.001), and MNSI score (*P* < 0.001).

**Figure 1:**
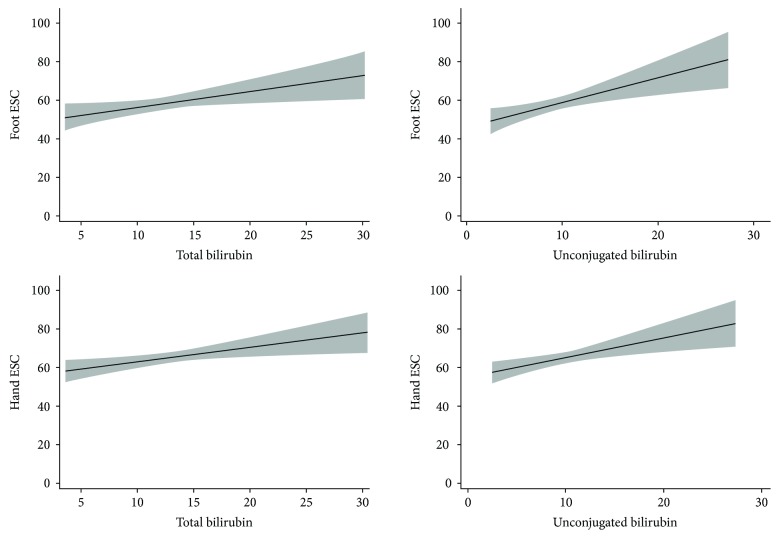
Multiple linear regression analysis of association between total and unconjugated bilirubin levels and hand and foot ESC levels detected by the SUDOSCAN.

Spearman correlation analysis demonstrated significant correlation coefficients between total bilirubin and ESC levels including hands (*r* = 0.165, *P* < 0.05) and feet (*r* = 0.122, *P* < 0.05) as well as between UCB and ESC levels including both hands (*r* = 0.172, *P* < 0.05) and feet (*r* = 0.175, *P* < 0.05). (Table 3) However, we did not detect any significant correlation between CB with hand or foot ESC levels. On multiple linear regression analysis, we found that higher total bilirubin level was independently associated with higher hand (*β* coefficient = 0.757, *P* < 0.05) and foot ESC levels (*β* coefficient = 0.757, *P* < 0.05). UCB but not CB level was independently correlated with hand ESC levels (*β* coefficient = 0.613, *P* < 0.05) and foot ESC levels (*β* coefficient = 0.764, *P* < 0.05); the result of which was consistent with previous spearman correlation analysis (as we can see in Table 4 and Figure 1).

## 4. Discussion

In the present study, we found that both TBil and UCBil levels were highly correlated with both hand and foot ESC levels detected by the SUDOSCAN; the result of which indicated that elevation of bilirubin level might have a protective role in the development of diabetic neuropathy.

Bilirubin (including TBil, UCBil, and CBil), a metabolite of heme and has been shown as a potent endogenous antioxidant, is traditional liver function index. Serum UCBil is carried by albumin to the liver where the hepatic enzyme UDP-glucuronyl transferase 1A1 converts UCBil to CBil. When TBil is in a normal range, higher CBil indicates hepatocellular injury.

In the year 2010, the National Health and Nutrition Examination Survey (NHANES) performed to evaluate the overall health and nutrition status of the United States population showed that increased TBil level was associated with 26% reduction in diabetes risk (OR = 0.74, 95% CI 0.64–0.88) [25]. Multivariate analysis results also confirmed this association (OR = 0.80, 95% CI 0.67–0.95) after adjusting for all diabetes risk factors. Additionally, several cross-sectional and cohort studies also reported an inverse association between serum TBil and T2DM [8, 26].

Apart from T2DM, there is also a growing body of literature which shows that higher total bilirubin levels are protective against cardiovascular disease, stroke, and peripheral arterial disease. In the year 2008, Fukui et al. [27] showed a significant inverse correlation between the serum bilirubin concentration and pulse wave velocity, while a significant positive correlation was found to the ankle brachial index in a subgroup of 386 patients. They also found that serum bilirubin level is associated with microalbuminuria and subclinical atherosclerosis in patients with type 2 diabetes. Concerning the role bilirubin might play in diabetic complications, evidence from a recent meta-analysis showed a negative nonlinear association between bilirubin concentration and the risk of diabetic complications (OR = 0.77, 95% CI 0.73–0.81) [9]. They found that there was a negative association between bilirubin concentration and the risk of diabetic nephropathy, diabetic retinopathy, and diabetic neuropathy indicating that bilirubin may play a protective role in the occurrence of diabetic complications. The possible underlying mechanism might be due to a potent antioxidant property of bilirubin by inhibiting lipid peroxidation and attenuating low-density lipoprotein (LDL) oxidation in diabetic patients. In recent years, the number of studies on the relationship between bilirubin concentration and the risk of diabetic complications has increased; some studies have indicated that high bilirubin concentration has a protective effect on diabetic complications, but some studies did not find this relationship. Age and race might be two main factors contributing to these inconsistent findings. However, among these related clinical studies, very few of them have focused on diabetic neuropathy. In the year 2014, Chung et al. [28] first showed that serum TBil levels were significantly associated with cardiovascular autonomic neuropathy (OR = 0.36; 95% CI 0.21–0.63 for the highest versus the lowest bilirubin tertile, *P* < 0.001) suggesting that TBil level might be inversely associated with the prevalence of cardiovascular autonomic neuropathy in patients with type 2 diabetes. Later in the year 2015, Kim et al. [29] also found that TBil level was inversely associated with the presence of DPN in 1207 patients aged more than 30 years with type 2 diabetes in Korea. In the study, they found that low serum bilirubin levels were significantly associated with DPN, independently of classic risk factors and other microvascular complications. In a study in the year 2015, Jian Liu et al. [30] found that unconjugated bilirubin mediated heme oxygenase-1-induced vascular benefits which suggested bilirubin as a potential therapeutic target for clinical intervention of diabetic vasculopathy.

In our study, we decided to use the SUDOSCAN as a tool for the early detection of diabetic neuropathy to evaluate the relationship between bilirubin and neuropathy. We detected a relatively high correlation between both hand and foot ESC levels with total and unconjugated bilirubin levels by using multiple linear regression analysis. However, we do have some limitations in this study. First, this cross-sectional study only provided a relationship but failed to provide any causal effect between bilirubin and the occurrence of peripheral small nerve dysfunction. Secondly, the sample size of this study is not large enough to be further divided according to HbA1c or levels of other clinical factors. Thirdly, in this study, we did not perform other diabetic microvascular complication including diabetic retinopathy and automatic cardiovascular disease to provide a comprehensive understanding of how bilirubin level correlates with all diabetic microvascular complications.

As far as we know, this study is a pilot study on understanding a possible relation between bilirubin level and ESC value which has been used to detect early peripheral small nerve fiber dysfunction caused by overproduction of oxidative products.

To conclude, high total and unconjugated serum bilirubin levels are inversely associated with DPN, independently of classic risk factors. Further investigations are necessary on the underlying mechanism that links bilirubin to neuroprotection and the prognostic significance of serum bilirubin on DPN as well as considering elevating bilirubin metabolism as a potential therapeutic target to ameliorate a variety of conditions.

## Figures and Tables

**Table 1:** Baseline characteristics of 265 T2DM patients enrolled in the study.

	Mean ± SD
Age (years)	59.64 ± 12.83
Male/female	163/102
Duration of T2DM (years)	9.20 ± 7.84
SBP (mmHg)	129.53 ± 14.07
DBP (mmHg)	80.28 ± 9.20
HbA1c (%)	8.67 ± 2.18
BMI (kg/m^2^)	24.53 ± 4.10
WHR	0.93 ± 0.07
ALT (U/L)	30.35 ± 20.77
AST (U/L)	22.97 ± 14.08
TG (mmol/L)	1.81 ± 1.93
CHO (mmol/L)	4.49 ± 1.23
LDL-C (mmol/L)	2.58 ± 0.88
HDL-C (mmol/L)	1.22 ± 2.91
Smokers (%)	76 (28.7%)
Alcohol consumption (%)	31 (11.7%)
Total bilirubin (*μ*mol/L)	12.54 ± 4.48
Conjugated bilirubin (*μ*mol/L)	3.34 ± 3.44
Unconjugated bilirubin (*μ*mol/L)	9.27 ± 4.81
MNSI score	3.04 ± 2.31
NSS score	3.07 ± 2.78
NDS score	2.89 ± 2.59
DPN by SUDOSDCAN (%)	148 (53.0%)
Foot ESC (*μ*S)	59.14 ± 21.95
Hand ESC (*μ*S)	59.51 ± 21.95

Data are means (SD) and percentage (%). T2DM: type 2 diabetes mellitus; BMI: body mass index; SBP: systolic blood pressure; DBP: diastolic blood pressure; DPN: diabetic peripheral neuropathy; WHR: waist-hip ratio; HbA1c: glycated hemoglobin; LDL-C: low-density lipoprotein cholesterol; HDL-C: high-density lipoprotein cholesterol; CHO: cholesterol; TG: triglyceride; ALT: alanine aminotransferase; AST: aspartate aminotransferase; TBil: total bilirubin; CBil: conjugated bilirubin; UCBil: unconjugated bilirubin; NSS: neuropathy symptom score; NDS: neuropathy disability score; MNSI: Michigan Neuropathy Screening Instrument; ESC: electrochemical skin conductance.

**Table 2:** Characteristics of 265 T2DM patients enrolled in the study divided by DPN diagnosed by the SUDOSCAN.

	DPN group (*N* = 148)	Non-DPN group (*N* = 117)	*P* value
Age (years)	60.53 ± 13.05	58.79 ± 12.21	0.257
Female (%)	89 (60.1%)	79 (67.5%)	0.902
Duration of T2DM (years)	10.25 ± 7.90	8.24 ± 7.61	0.048^∗^
SBP (mmHg)	130.62 ± 14.57	128.17 ± 13.82	0.158
DBP (mmHg)	80.71 ± 9.51	80.06 ± 8.29	0.558
HbA1c (%)	8.62 ± 2.00	8.76 ± 2.39	0.634
BMI (kg/m^2^)	24.36 ± 4.05	24.59 ± 4.09	0.642
WHR	0.94 ± 0.07	0.92 ± 0.06	0.030^∗^
ALT (U/L)	30.71 ± 20.85	30.07 ± 20.89	0.800
AST (U/L)	23.38 ± 13.90	22.66 ± 14.39	0.672
CHO (mmol/L)	4.39 ± 1.05	4.63 ± 1.39	0.124
TG (mmol/L)	1.56 ± 0.88	2.11 ± 2.64	0.130
LDL-C (mmol/L)	2.61 ± 0.89	2.56 ± 0.87	0.742
HDL-C (mmol/L)	1.06 ± 0.39	1.05 ± 0.36	0.809
Smokers (%)	89 (89/133)	80 (80/115)	0.864
Alcohol consumption (%)	115 (87.8%)	99 (86.1%)	0.708
TBil (*μ*mol/L)	13.90 ± 9.78	16.33 ± 14.83	0.105
CBil (*μ*mol/L)	5.12 ± 9.76	6.84 ± 16.14	0.279
UCBil (*μ*mol/L)	8.97 ± 4.47	9.48 ± 6.05	0.423
MNSI score	3.54 ± 2.46	2.44 ± 1.96	0.000^∗∗^
NSS score	3.30 ± 2.71	2.29 ± 2.26	0.000^∗∗^
NDS score	3.38 ± 2.71	2.29 ± 2.26	0.000^∗∗^
Foot ESC (*μ*S)	44.98 ± 19.89	75.68 ± 9.05	0.000^∗∗^
Hand ESC (*μ*S)	47.75 ± 17.54	73.27 ± 9.67	0.000^∗∗^

Data are means (SD) and percentage (%). T2DM: type 2 diabetes mellitus; BMI: body mass index; SBP: systolic blood pressure; DBP: diastolic blood pressure; DPN: diabetic peripheral neuropathy; WHR: waist-hip ratio; HbA1c: glycated hemoglobin; LDL-C: low-density lipoprotein cholesterol; HDL-C: high-density lipoprotein cholesterol; CHO: cholesterol; TG: triglyceride; ALT: alanine aminotransferase; AST: aspartate aminotransferase; TBil: total bilirubin; CBil: conjugated bilirubin; UCBil: unconjugated bilirubin; NSS: neuropathy symptom score; NDS: neuropathy disability score; MNSI: Michigan Neuropathy Screening Instrument; ESC: electrochemical skin conductance. *P* value was calculated after adjustment for age, sex except for itself. ^∗^*P* < 0.05; ^∗∗^*P* < 0.01.

**Table 3:** Spearman correlation between traditional screening scores including NSS, NDS, and MNSI and hand and foot ESC level detected by the SUDOSCAN and bilirubin level (total, unconjugated, and conjugated).

	Total bilirubin (TBil)	Unconjugated bilirubin (UCBil)	Conjugated bilirubin (CBil)
NSS	−0.003	0.052	−0.092
NDS	−0.046	−0.067	0.027
MNSI	0.024	0.075	−0.097
Hand ESC	0.165^∗^	0.172^∗^	0.053
Foot ESC	0.122^∗^	0.175^∗^	−0.050

TBil: total bilirubin; CBil: conjugated bilirubin; UCBil: unconjugated bilirubin; NSS: neuropathy symptom score; NDS: neuropathy disability score; MNSI: Michigan Neuropathy Screening Instrument; ESC: electrochemical skin conductance. ^∗^*P* < 0.05.

**Table 4:** Multiple linear regression analysis of association between bilirubin level and NSS, NDS, and MNSI scores and ESC levels detected by the SUDOSCAN.

	Total bilirubin	*P* value	Unconjugated bilirubin	*P* value	Conjugated bilirubin	*P* value
NSS	−0.018 (−0.103 to 0.067)	0.680	0.003 (−0.081 to 0.087)	0.939	−0.056 (−0.191 to 0.078)	0.411
NDS	−0.029 (−0.111 to 0.054)	0.494	−0.056 (−0.137 to 0.025)	0.175	0.035 (−0.096 to 0.165)	0.596
MNSI	0.014 (−0.031 to 0.059)	0.537	0.010 (−0.034 to 0.054)	0.659	−0.023 (−0.146 to 0.100)	0.712
Hand ESC	0.757 (0.159 to 1.355)	0.013^∗^	0.613 (0.018 to 1.208)	0.044^∗^	0.110 (−0.827 to 1.047)	0.817
Foot ESC	0.757 (0.066 to 1.449)	0.032^∗^	0.764 (0.076 to 1.451)	0.030^∗^	−0.254 (−1.329 to 0.821)	0.642

NSS: neuropathy symptom score; NDS: neuropathy disability score; MNSI: Michigan Neuropathy Screening Instrument; ESC: electrochemical skin conductance. ^∗^*P* < 0.05.

## Abbreviations

T2DM: Type 2 diabetes mellitus
BMI: Body mass index
SBP: Systolic blood pressure
DBP: Diastolic blood pressure
DPN: Diabetic peripheral neuropathy
WHR: Waist-hip ratio
HbA1c: Glycated hemoglobin
LDL-C: Low-density lipoprotein cholesterol
HDL-C: High-density lipoprotein cholesterol
CHO: Cholesterol
TG: Triglyceride
ALT: Alanine aminotransferase
AST: Aspartate aminotransferase
TBil: Total bilirubin
CBil: Conjugated bilirubin
UCBil: Unconjugated bilirubin
NSS: Neuropathy symptom score
NDS: Neuropathy disability score
MNSI: Michigan Neuropathy Screening Instrument
ESC: Electrochemical skin conductance.

## References

[1] Roland Stocker Y. Y., McDonagh A. F., Glazer A. N., Ames B. N. (1987). Bilirubin is an antioxidant of possible physiological importance. *Science*.

[2] Hendrickse M. T., Triger D. R. (1993). Autonomic and peripheral neuropathy in primary biliary cirrhosis. *Journal of Hepatology*.

[3] Ko G. T. C. J., Woo J., Lau E. (1996). Serum bilirubin and cardiovascular risk factors in a Chinese population. *Journal of Cardiovascular Risk*.

[4] Schwertner H. A., Jackson W. G., Tolan G. (1994). Association of low serum concentration of bilirubin with increased risk of coronary artery disease. *Clinical Chemistry*.

[5] Mayer M. (2000). Association of serum bilirubin concentration with risk of coronary artery disease. *Clinical Chemistry*.

[6] Lin J. P., Vitek L., Schwertner H. A. (2010). Serum bilirubin and genes controlling bilirubin concentrations as biomarkers for cardiovascular disease. *Clinical Chemistry*.

[7] Perlstein T. S., Pande R. L., Beckman J. A., Creager M. A. (2008). Serum total bilirubin level and prevalent lower-extremity peripheral arterial disease: National Health and Nutrition Examination Survey (NHANES) 1999 to 2004. *Arteriosclerosis, Thrombosis, and Vascular Biology*.

[8] Wang J., Li Y., Han X. (2017). Serum bilirubin levels and risk of type 2 diabetes: results from two independent cohorts in middle-aged and elderly Chinese. *Scientific Reports*.

[9] Zhu B., Wu X., Bi Y., Yang Y. (2017). Effect of bilirubin concentration on the risk of diabetic complications: a meta-analysis of epidemiologic studies. *Scientific Reports*.

[10] Liu M., Li Y., Li J., Lv X., He Y. (2017). Elevated serum total bilirubin levels are negatively associated with major diabetic complications among Chinese senile diabetic patients. *Journal of Diabetes and its Complications*.

[11] Yasuda M., Kiyohara Y., Wang J. J. (2011). High serum bilirubin levels and diabetic retinopathy: the Hisayama study. *Ophthalmology*.

[12] Karuppannasamy D., Venkatesan R., Thankappan L., Andavar R., Devisundaram S. (2017). Inverse association between serum bilirubin levels and retinopathy in patients with type 2 diabetes mellitus. *Journal of Clinical and Diagnostic Research*.

[13] Cho H. C. (2011). The relationship among homocysteine, bilirubin, and diabetic retinopathy. *Diabetes & Metabolism Journal*.

[14] Toya K., Babazono T., Hanai K., Uchigata Y. (2014). Association of serum bilirubin levels with development and progression of albuminuria, and decline in estimated glomerular filtration rate in patients with type 2 diabetes mellitus. *Journal of Diabetes Investigation*.

[15] Tesfaye S., Boulton A. J., Dyck P. J. (2010). Diabetic neuropathies: update on definitions, diagnostic criteria, estimation of severity, and treatments. *Diabetes Care*.

[16] Vinik A. I., Nevoret M. L., Casellini C. (2015). The new age of sudomotor function testing: a sensitive and specific biomarker for diagnosis, estimation of severity, monitoring progression, and regression in response to intervention. *Frontiers in Endocrinology*.

[17] Muller G., Parfentyeva E., Olschewsky J., Bornstein S. R., Schwarz P. E. (2013). Assessment of small fiber neuropathy to predict future risk of type 2 diabetes. *Primary Care Diabetes*.

[18] Casellini C. M., Parson H. K., Richardson M. S., Nevoret M. L., Vinik A. I. (2013). Sudoscan, a noninvasive tool for detecting diabetic small fiber neuropathy and autonomic dysfunction. *Diabetes Technology & Therapeutics*.

[19] Yajnik C. S., Kantikar V. V., Pande A. J., Deslypere J. P. (2012). Quick and simple evaluation of sudomotor function for screening of diabetic neuropathy. *ISRN Endocrinology*.

[20] Mao F., Liu S., Qiao X. (2017). Sudoscan is an effective screening method for asymptomatic diabetic neuropathy in Chinese type 2 diabetes mellitus patients. *Journal of Diabetes Investigation*.

[21] Smith A. G., Lessard M., Reyna S., Doudova M., Singleton J. R. (2014). The diagnostic utility of Sudoscan for distal symmetric peripheral neuropathy. *Journal of Diabetes and its Complications*.

[22] Abbott C. A. C. A. L., Ashe H., Bath S. (2002). The North-West Diabetes Foot Care Study: incidence of, and risk factors for, new diabetic foot ulceration in a community-based patient cohort. *Diabetic Medicine*.

[23] Xiong Q., Lu B., Ye H., Wu X., Zhang T., Li Y. (2015). The diagnostic value of neuropathy symptom and change score, neuropathy impairment score and Michigan neuropathy screening instrument for diabetic peripheral neuropathy. *European Neurology*.

[24] Moghtaderi A., Bakhshipour A., Rashidi H. (2006). Validation of Michigan neuropathy screening instrument for diabetic peripheral neuropathy. *Clinical Neurology and Neurosurgery*.

[25] Cheriyath P., Gorrepati V. S., Peters I. (2010). High Total bilirubin as a protective factor for diabetes mellitus: an analysis of NHANES data from 1999 - 2006. *Journal of Clinical Medicine Research*.

[26] Abbasi A., Deetman P. E., Corpeleijn E. (2015). Bilirubin as a potential causal factor in type 2 diabetes risk: a Mendelian randomization study. *Diabetes*.

[27] Fukui M., Tanaka M., Shiraishi E. (2008). Relationship between serum bilirubin and albuminuria in patients with type 2 diabetes. *Kidney International*.

[28] Chung J. O., Cho D. H., Chung D. J., Chung M. Y. (2014). Physiological serum bilirubin concentrations are inversely associated with the prevalence of cardiovascular autonomic neuropathy in patients with type 2 diabetes. *Diabetic Medicine*.

[29] Kim E. S., Lee S. W., Mo E. Y., Moon S. D., Han J. H. (2015). Inverse association between serum total bilirubin levels and diabetic peripheral neuropathy in patients with type 2 diabetes. *Endocrine*.

[30] Jian Liu L. W., Tian X. Y., Liu L. (2015). Unconjugated bilirubin mediates heme oxygenase-1-induced vascular benefits in diabetic mice. *Diabetes*.

